# Obesity and metabolic disease in migrants: a role for the gut microbiome?

**DOI:** 10.3389/fcdhc.2025.1745885

**Published:** 2026-01-14

**Authors:** Babatunde Fasipe, Ismail Laher

**Affiliations:** 1 Department of Pharmacology and Therapeutics, Faculty of Basic Clinical Sciences, Bowen University, Iwo, Nigeria; 2 Department of Anesthesiology, Pharmacology and Therapeutics, Faculty of Medicine, The University of British Columbia, Vancouver, BC, Canada

**Keywords:** acculturation, circadian rhythm, gut microbiome, health disparities, insulin resistance, migration, obesity, mTOR signaling

## Abstract

Migration, while often motivated by safety, education, or economic opportunity, often heightens the risk of obesity and metabolic syndrome. Resettlement in industrialized nations is associated with sedentary lifestyles, irregular sleep schedules, and Westernized dietary patterns rich in ultra-processed, high-fat, and high-sugar foods. These changes disrupt metabolic homeostasis through endocrine and circadian dysregulation, promoting insulin resistance, visceral adiposity, and systemic inflammation. Migration alters the composition and diversity of the gut microbiome, suggesting that the characteristics of the microbiome could be important in linking migration to changes in health outcomes after resettlement. However, the precise mechanisms underlying these microbiome-mediated effects remain poorly understood. We propose that a dynamic metabolic interface is reshaped via a rapid “microbiome acculturation”, which is a process by which the gut microbiome rapidly adapts to a new cultural and environmental milieu, such as caused by migration, shifting from traditional, fiber-rich microbial profiles to Westernized, *Bacteroides*-dominant communities associated with metabolic dysfunction. This is characterized by the depletion of fiber-fermenting *Prevotella* and enrichment of *Bacteroides* species, leading to reduced short-chain fatty acid production, impaired gut barrier function, and increased endotoxemia. Dietary transitions, chronic psychosocial stress, circadian disruption to night-shift work, and reduced physical activity experienced by immigrants reshapes gut microbial composition and function to a pro-inflammatory milieu and enhancing insulin resistance. Thus, gut dysbiosis serves as both a biomarker and mechanistic driver of post-migration metabolic deterioration, integrating dietary, behavioral, and environmental stressors into a unified pathogenic pathway. Effective prevention should target the gut–brain–metabolic axis using multidimensional strategies: restoring microbial diversity using high-fiber, prebiotic, and probiotic nutrition; promoting physical activity and circadian alignment; and addressing social determinants of health such as work patterns, food access, and acculturation stress.

## 1 Introduction

Migration is a hallmark of times of uncertainty and conflict. Many people migrate in search of greater safety, improved education, family reunification, political stability, and economic opportunities (1–3). The UN DESA and the OECD emphasize labor migration as the dominant flow toward high-income nations (4, 5). While relocation can bring safety and prosperity, it also introduces unintended health consequences, chief among them being obesity and related metabolic disorders (6). Exposures to calorie-dense foods and sedentary lifestyles in industrialized settings contrast sharply with the more active and balanced nutritional choices of the environments they emigrated from (7). Epidemiological studies consistently indicate that higher body mass index (BMI) and prevalence of type 2 diabetes and cardiovascular disease correlate with the duration of relocation to more industrialized nations, reflecting an environment-driven acceleration of metabolic dysregulation (8–10). Although migration is a powerful driver of metabolic disorders through lifestyle and environmental adaptations, the role of the gut microbiome in shaping these outcomes remains largely underexplored. An extensive body of research demonstrates that the human gut hosts trillions of microorganisms that play integral roles in maintaining physiological and metabolic homeostasis. This complex ecosystem, comprising bacteria, fungi, viruses, and archaea, exists in a symbiotic relationship with the human host, influencing numerous aspects of health and disease. Disruptions in the composition or function of this microbial community, known as dysbiosis, have been linked to an increased susceptibility to several chronic conditions, including diabetes, obesity, kidney disease, and inflammatory bowel disease (11–13).

Extensive analyses of existing studies indicate that migration induces significant shifts in the composition of the gut microbiome. These alterations reflect adaptations to new environmental, dietary, and lifestyle conditions following relocation (14–17) (**Table 1**). We proposed that acculturation associated with dietary shifts, altered physical activity patterns, and psychosocial stress linked to migration, profoundly influences gut microbial composition and metabolic signaling, resulting in susceptibility of migrants to obesity, insulin resistance, and type 2 diabetes. Understanding the microbiome’s role as a biological interface between migration-induced lifestyle transitions and metabolic disease could provide novel insights into preventive and therapeutic strategies targeting this vulnerable population (18–20).

**Table 1:** Studies of migration and gut microbiome composition.

Citation	Population & migration	Host country	Design	N (approx.)	Main microbiome change after migration	Time scale
Vangay et al. (15),	Hmong & Karen adults migrating from SE Asia to the U.S.; first-gen & second-gen comparison	United States	Cross-sectional + longitudinal (stool metagenomics, diet)	n≈514	Loss of alpha-diversity; shift from Prevotella-rich to Bacteroides-dominant; loss of fiber-degrading functions	Within months; progression with longer residence
Kaplan et al. (16),	Hispanic/Latino adults; US-born vs foreign-born; relocation across regions	United States	Population cohort analysis	n≈1674	Microbiome composition associated with geographic relocation, birthplace, and obesity; US-born vs foreign-born differences	Cross-sectional across nativity and relocation
Verhaar et al., (17)	Ghanaian adults: rural Ghana, urban Ghana, and Ghanaian migrants in Amsterdam	Netherlands (comparison with Ghana)	Multi-site cohort (16S rRNA); gradient along migration axis	n≈1,177	Clear shift in community composition and diversity from rural→urban→Amsterdam; taxa differences partly explained by diet; links to cardiometabolic markers	Cross-sectional along migration gradient

Economic survival on resettlement often entails engaging in multiple jobs and night-shift work, which disrupt circadian rhythms, degrade sleep, and limit time for physical activity (21, 22), which coupled with increased caloric exposure, creates a potent triad for obesity and cardiometabolic disease (23, 24). Obesity rates increase with duration of residence (25, 26). Seminal studies show obesity increases markedly within 10–15 years of arrival, approaching or exceeding native-born levels (4), with parallel patterns in Canada and Europe (25). Beyond behavior, migration reshapes diet, stress, and socioeconomic context, where traditional plant-rich diets are replaced by Westernized, energy-dense foods (27, 28), while acculturative stress, discrimination, and economic hardship further accelerate weight gain (29, 30). This erosion of the “healthy immigrant effect” exemplifies the acculturation paradox.

## 2 mTOR as a regulator of gut microbiome homeostasis

**Figure 1:**
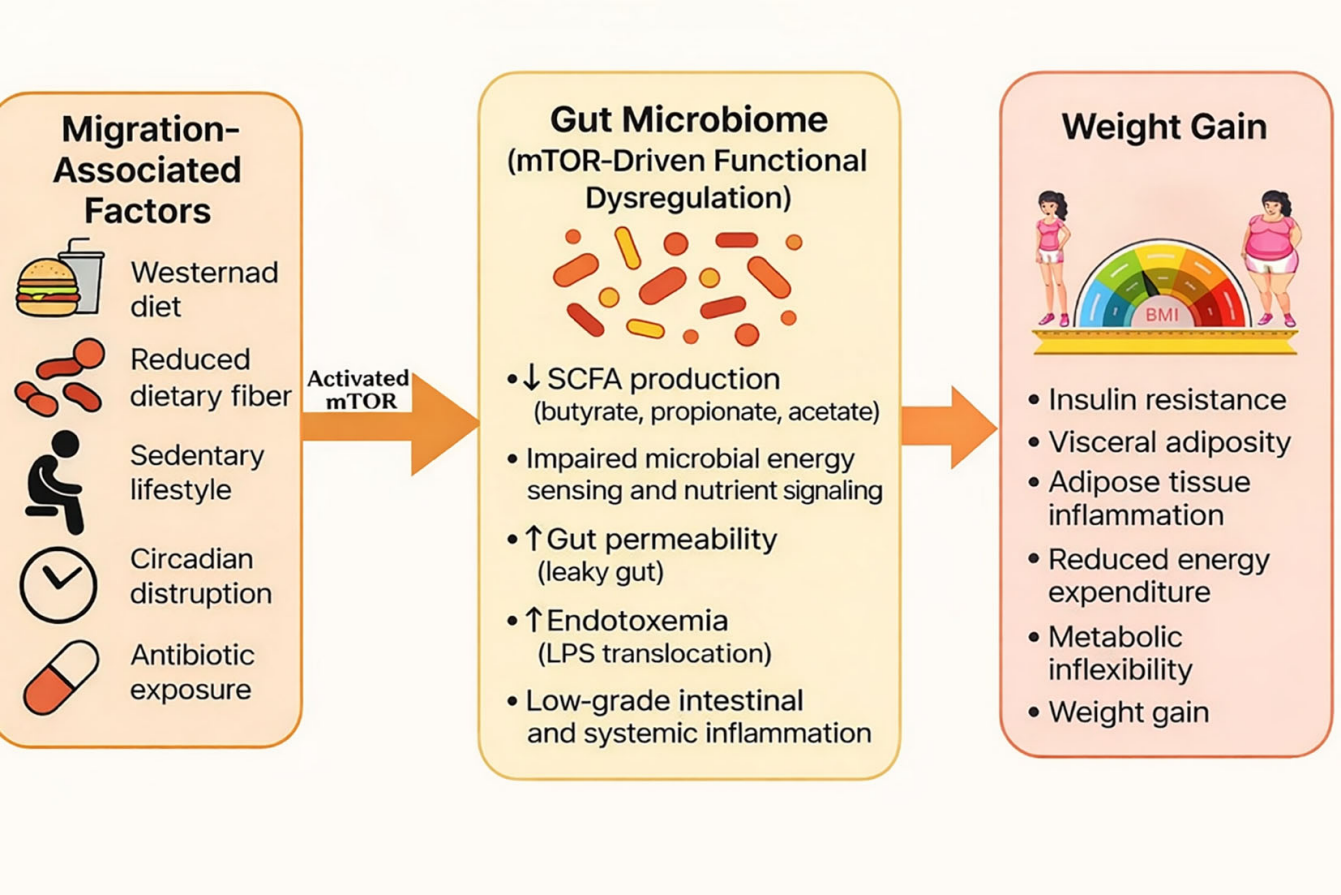
Migration-induced weight gain mediated by gut microbiome–mTOR crosstalk. Migration is associated with lifestyle and environmental changes, including adoption of Westernized diets, reduced dietary fiber intake, increased sedentary behavior, circadian disruption, and exposure to antibiotics. These factors drive rapid remodeling of the gut microbiome, characterized by reduced microbial diversity, depletion of Prevotella and other short-chain fatty acid (SCFA)–producing taxa, dominance of Bacteroides species, altered SCFA profiles (butyrate, propionate, acetate), increased gut permeability, and metabolic endotoxemia (lipopolysaccharide, LPS). Microbiome-derived metabolites and inflammatory signals converge on nutrient-sensing pathways, particularly mammalian target of rapamycin (mTOR), promoting insulin resistance, visceral adiposity, adipose tissue inflammation, reduced energy expenditure, metabolic inflexibility, and progressive weight gain. This schematic highlights mTOR as a central integrative node linking migration-associated environmental exposures to microbiome-driven metabolic dysfunction.

The mammalian target of rapamycin (mTOR) is a central nutrient- and energy-sensing kinase that integrates signals from glucose, amino acids, insulin, growth factors, and inflammatory cues to regulate cellular growth, metabolism, and survival (31, 32). Within the gastrointestinal tract, mTOR signaling has a pivotal role in intestinal epithelial turnover, barrier integrity, autophagy, and innate immune defense, thereby helping to define the ecological environment of the gut microbiome (33). When nutrient availability is balanced, tightly regulated mTOR activity supports mucosal homeostasis and symbiotic host–microbe interactions. However, chronic overactivation of mTOR, such as that induced by excess caloric intake, hyperinsulinemia, and Westernized dietary patterns, suppresses autophagy and compromises epithelial antimicrobial function (31, 34) (**Figure 1**). This dysregulated mTOR state creates conditions that favor microbial dysbiosis, low-grade inflammation, and increased metabolic efficiency, thereby linking host nutrient signaling to gut microbiome remodeling and downstream weight gain (35, 36). Dietary acculturation and lifestyle changes in immigrants promote chronic activation of mTOR signaling coupled with rapid remodeling of the gut microbiome. These interacting host–microbial pathways reduce metabolic flexibility, suppress adaptive energy expenditure, and favor energy storage, and increased risk of weight gain.

This review examines five interconnected domains that collectively determine metabolic risk following migration: (1) reduced physical activity (2) dietary transitions, (3) circadian disruption from night-shift employment (4), psychosocial stress and cultural adaptation, and (5) gut microbiome alterations, through the activation of mTOR as central integrator that may link these factors. Each domain interacts through behavioral, hormonal, and microbial pathways through mTOR hyperactivation which translates environmental and lifestyle changes into metabolic outcomes. These mechanisms can collectively explain the progressive increases in rates of obesity and metabolic disorders after migration and highlight key entry points for culturally responsive prevention and intervention strategies.

### 2.1 Decreases in physical activity

Movement is embedded in daily activities such as agricultural labor, market trading, carrying water, and walking between towns, which most migrants engaged in for many years before they relocate (37). Migration exposes newly arrived settlers to increased mechanization, urbanization, and motorized transport in place of manual effort. Desk-based work, long commutes, and digital tools sharply reduce baseline activity further, and multiple jobs and caregiving limits time or energy for structured exercise (38). Inactivity lowers mitochondrial efficiency, impairs glucose uptake, and increases insulin resistance, key factors that can lead to type 2 diabetes and cardiovascular disease (39) (**Table 2**). Suppressed skeletal-muscle oxidative capacity and reduced myokine signaling limit anti-inflammatory and vasoprotective mechanisms. Thus, the drop in habitual movement after migration represents a foundational shift in energy metabolism that propels obesity and its comorbidities.

**Table 2:** Sociocultural and environmental domains influencing obesity risk among migrants and potential interventions.

Domain	Mechanisms contributing to obesity	References	Potential intervention
Decreases in Physical Activity	Diminished mitochondrial efficiency, impaired glucose uptake, increased insulin resistance → T2D/CVD risk	(31–34)	Community-based activity programs; culturally adapted exercise; workplace movement policies
Dietary Transitions and Nutrition Acculturation	Westernized diets with ↑ refined carbs/fats/sugars, ↓ fiber; oxidative stress; microbiome shift with ↓ Prevotella and ↓ SCFAs	(40–46)	Culturally tailored nutrition education; greater access to fresh produce; support for healthy traditional dietary practices
Work Patterns & Circadian Disruption	Irregular/night work desynchronizes body clocks; worsens glucose tolerance, lipid handling; leptin↓ ghrelin/cortisol↑; visceral adiposity, insulin resistance	(47–67)	Limit prolonged shift work; optimize light exposure; improve sleep hygiene; align meals with circadian timing
Psychosocial Stress & Cultural Adaptation	Chronic stress activates HPA axis; ↑ cortisol/IL-6/TNF-α; visceral fat; emotional eating	(64, 68–74)	Stress-management; social support; culturally sensitive counseling; workplace equity
Impact on Gut Microbiome	Post-migration ↓ diversity; Prevotella→Bacteroides shift; ↑ energy harvest, ↓ SCFA signaling, ↑ permeability and inflammation	(75–84)	Fiber-rich diets, fermented foods, prebiotics/probiotics; preserve traditional foodways

This table summarizes five key domains through which migration-related lifestyle changes contribute to obesity and metabolic dysfunction: reduced physical activity, Western diets, circadian disruption, psychosocial stress, and gut microbiome alterations, that collectively impair mitochondrial efficiency, promote inflammation, and increase insulin resistance. The final column highlights potential interventions, including culturally appropriate exercise programs, nutrition education, circadian-aligned work schedules, psychosocial support, and dietary strategies emphasizing fiber-rich and fermented foods to preserve gut microbial diversity and metabolic health.

**Figure 2:**
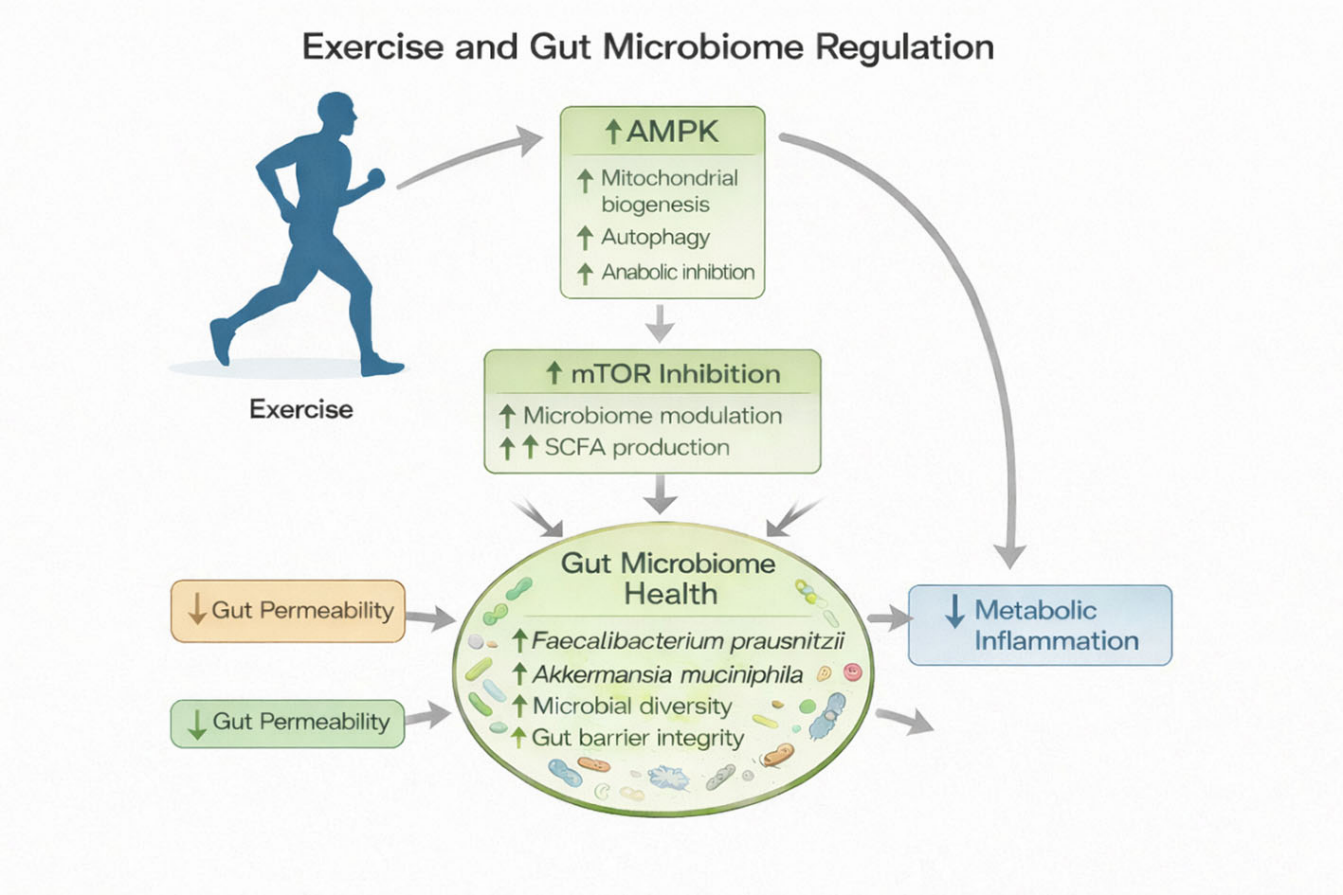
Exercise–mTOR–gut microbiome axis in metabolic regulation. Regular physical activity directly activates AMP-activated protein kinase (AMPK), promoting mitochondrial biogenesis and autophagy while suppressing anabolic signaling. AMPK activation leads to inhibition of mammalian target of rapamycin (mTOR), resulting in favorable modulation of the gut microbiome, including increased short-chain fatty acid (SCFA) production. These molecular adaptations enhance gut microbiome health by increasing beneficial taxa (e.g., Faecalibacterium prausnitzii and Akkermansia muciniphila), improving microbial diversity and gut barrier integrity, and reducing intestinal permeability. Collectively, these effects contribute to reduced metabolic inflammation and improved metabolic homeostasis.

Regular physical activity improves the gut microbiome by increasing microbial diversity and enhancing the abundance of beneficial bacteria such as *Akkermansia muciniphila*, *Faecalibacterium prausnitzii*, and other short-chain fatty acid (SCFA)–producing species, including acetate, propionate, and butyrate, which are generated by anaerobic fermentation of indigestible dietary fibers and resistant starches by commensal gut microbes in the colon. These metabolites serve as key signaling molecules and energy substrates that confer multiple metabolic advantages: butyrate fuels colonocytes and strengthens intestinal barrier integrity; propionate participates in hepatic gluconeogenesis and appetite regulation, and also stimulates the release of PYY and GLP-1 from human colonic cells preventing weight gain in overweight adult humans (85). Acetate contributes to lipid metabolism and peripheral energy balance, while elevation of butyrate levels following exercise also limits lipopolysaccharide (LPS) translocation, and mitigates inflammatory signaling (86). Concurrently, activation of the AMPK–Nrf2 antioxidant axis by exercise and microbial metabolites attenuates oxidative stress across intestinal, hepatic, and skeletal muscle tissues (40, 87) (**Figure 2**). It also increases mTOR inhibition to prevent gut microbiome dysregulation. Collectively, these adaptations restore redox homeostasis, dampen systemic inflammation, and promote metabolic resilience characterized by improved insulin sensitivity and lipid metabolism. Collectively, SCFAs modulate host metabolism by activating G-protein–coupled receptors (GPR41 and GPR43), promote anti-inflammatory cytokine production, improves insulin sensitivity, and enhances mitochondrial function. Thus, exercise-induced enrichment of SCFA-producing bacteria supports metabolic homeostasis and protects against obesity-related insulin resistance and systemic inflammation (41–44).

Thus, exercise-modulated changes in the microbiome contributes to improved glucose regulation, lipid metabolism, immune function, and even brain health via the gut-brain axis. Moderate aerobic exercise produces the most consistent benefits, while extreme endurance training may temporarily disrupt gut integrity (45). Recent evidence suggests that the combined effects of exercise, dietary modulation, and gut microbial adaptations act synergistically to enhance metabolic health and performance outcomes beyond the benefits achieved by any single intervention alone (46).

### 2.2 Dietary transitions

Dietary changes are among the most rapid and consequential shifts after resettlement. Traditional agrarian and community-based diets emphasize vegetables, legumes, fruits, and whole grains prepared from minimally processed ingredients (47). Synchronized with seasonality and communal eating, these patterns correlate with lower obesity, diabetes, and cardiovascular risk (48, 49). Post-migration, access, cost, time limitations, and cultural adaptation favor Westernized patterns that are high in refined carbohydrates, processed meats, sugary beverages, and saturated fats (50). The increased intake of calorie-dense, high-fat, and refined-carbohydrate foods, promotes chronic activation of nutrient-sensing pathways, particularly mTOR signaling. Sustained mTOR activation links these dietary changes to impaired metabolic flexibility, altered gut microbiome composition, and increased risk of obesity and metabolic disease (51). Food environments in low-income urban areas (commonly inhabited by migrants) are dominated by fast food and convenience outlets and fewer outdoor spaces for recreational activities such as walking etc., further limit healthy choices (52). Long hours and tight budgets reinforce reliance on inexpensive, calorie-dense items, while the perceived modernity of previously scarce foods erodes traditional practices.

A diet rich in diverse, minimally processed plant foods, such as whole grains, legumes, fruits, vegetables, nuts, and seeds, increases gut microbiome diversity and overall metabolic health. This dietary pattern, often commonly practiced by migrants before relocation to Westernized societies, provides fermentable fibers that serve as substrates for beneficial gut bacteria, promoting the production of SCFAs to strengthen the gut barrier, enhance insulin sensitivity, and reduce systemic inflammation. However, upon migration, a shift toward Western diets that are high in refined carbohydrates, animal fats, and processed foods often diminishes microbial diversity and increases the risk of obesity and metabolic disease (53, 54).

In summary, migration often precipitates a profound nutritional transition that reshapes both metabolic health and gut microbial ecology. While traditional, plant-based diets that are rich in fiber and minimal consumption of processed foods promote microbial diversity and metabolic resilience, while post-migration adoption of Westernized dietary habits, driven by accessibility, convenience, affordability, and acculturation pressures, erodes these benefits. The resulting decline in beneficial SCFA, producing bacteria, coupled with increased consumption of refined and high-fat foods, contributes to heightened risks of obesity, insulin resistance, and cardiovascular disease. Addressing these challenges requires culturally sensitive nutritional strategies that preserve traditional dietary strengths while promoting sustainable, microbiome-friendly eating patterns in resettled populations.

### 2.3 Immigrants and night-shift work

Migrants often lack “local experience” and face systemic barriers to accreditation of professional qualifications, leading to many migrants being disproportionately represented in occupations that require night-shift or rotating-shift work, such as healthcare, transportation, manufacturing, and service industries. The economic pressures many migrants face due to limited employment options available to them, and the demand for around-the-clock labor, reduces access to better lifestyles and work opportunities. This increased exposure to nocturnal work profoundly disrupts their circadian rhythms, particularly the synchronization between central (light-entrained) and peripheral (feeding-entrained) clocks. Night-shift workers often consume meals (often unhealthy foods) during the biological night, when metabolic processes such as glucose tolerance, lipid oxidation, and insulin sensitivity are at their lowest (55, 56). This circadian misalignment of eating habits leads to impaired nutrient metabolism, altered gut microbiome rhythmicity, and increased risk of weight gain, insulin resistance, and cardiometabolic disorders. Moreover, irregular meal timing and frequent snacking to maintain alertness at night further exacerbates desynchronization between the body’s internal clocks and external cues. Consequently, migrants engaged in night-shift work face compounded metabolic risks arising from both social determinants and physiological disruption of circadian eating patterns (57–60).

The gut microbiome exhibits remarkable diurnal oscillations that are tightly linked to the host’s feeding and fasting cycles, influencing energy balance and metabolic health. These rhythmic fluctuations in microbial composition and function help coordinate nutrient absorption, SCFA production, and bile acid metabolism, thereby shaping systemic metabolic responses. However, the precise mechanisms underlying this bidirectional relationship remain incompletely understood. Eating during night shift work disrupts normal feeding–fasting rhythms and dampens the cyclical oscillations of gut microbial taxa, leading to metabolic inflexibility and weight gain. Conversely, time-restricted feeding (TRF), which consolidates food intake to the nocturnal phase, partially restores microbial rhythmicity and protects against obesity and metabolic disorders. TRF also enriches bacterial species that enhance host metabolic efficiency and reduce inflammation. Collectively, these findings suggest that the temporal pattern of food intake, along with dietary composition, profoundly affects the gut microbiome’s diurnal dynamics and its contribution to host metabolism. Consequently, both feeding timing and sample collection time are critical variables in studies assessing the microbiome’s role in metabolic regulation (61–64).

#### 2.3.1 Circadian rhythms and metabolic health

The circadian rhythm exerts a profound influence on the daily oscillations in gene expression, protein synthesis, metabolism, and cellular signaling. At its core lies a transcription–translation feedback loop involving the clock genes CLOCK, BMAL1, PER, and CRY, which regulate each other in roughly 24-hour cycles (65). This molecular clock drives rhythmic changes in chromatin structure, transcription factor activity, and noncoding RNA expression, resulting in time-dependent regulation of nearly half of the genome. Through interactions with epigenetic modifiers such as SIRT1 and histone acetyltransferases, circadian rhythms align cellular processes such as DNA repair, cell cycle progression, and energy metabolism with the environmental light–dark cycle (66, 67).

Night-shift work profoundly disrupts the circadian system and has been associated with increased risk of metabolic, gastrointestinal, cardiovascular, and neurocognitive disorders. Alterations in gut microbiota composition and function may serve as a key intermediary linking circadian disruption to these adverse health outcomes (68). Disruption of the circadian rhythm and alterations in the gut microbiome are interconnected through a bidirectional communication network encompassing the nervous, immune, and endocrine systems, collectively known as the gut–brain–circadian axis. When circadian rhythms are misaligned, such as during night-shift work or irregular feeding schedules, the gut microbiome loses its diurnal rhythmicity, resulting in compositional shifts and altered microbial metabolism. These changes disrupt the production of SCFAs and bile acid signaling, which in turn promote systemic inflammation, oxidative stress, and metabolic dysregulation (69, 70). Conversely, the gut microbiota modulates host circadian rhythms by sending feedback signals through microbial metabolites, cytokine-mediated immune pathways, and vagal nerve activation, influencing central and peripheral clock gene expression. This reciprocal regulation forms a feedback loop, where circadian misalignment alters microbial activity, and microbial disturbances that further reinforce circadian disruption, ultimately contributing to metabolic syndrome, obesity, and glucose intolerance (64, 71).

Disruption of circadian rhythm, such as from night-shift work or irregular sleep, leads to oxidative stress, insulin resistance, inflammation, and increased susceptibility to chronic diseases including obesity, type 2 diabetes, cardiovascular disorders, and cancer (72–74). Important in this context are recent epidemiological data supporting a bidirectional relationship between sleep disorders and obesity (75, 76).

**Figure 3:**
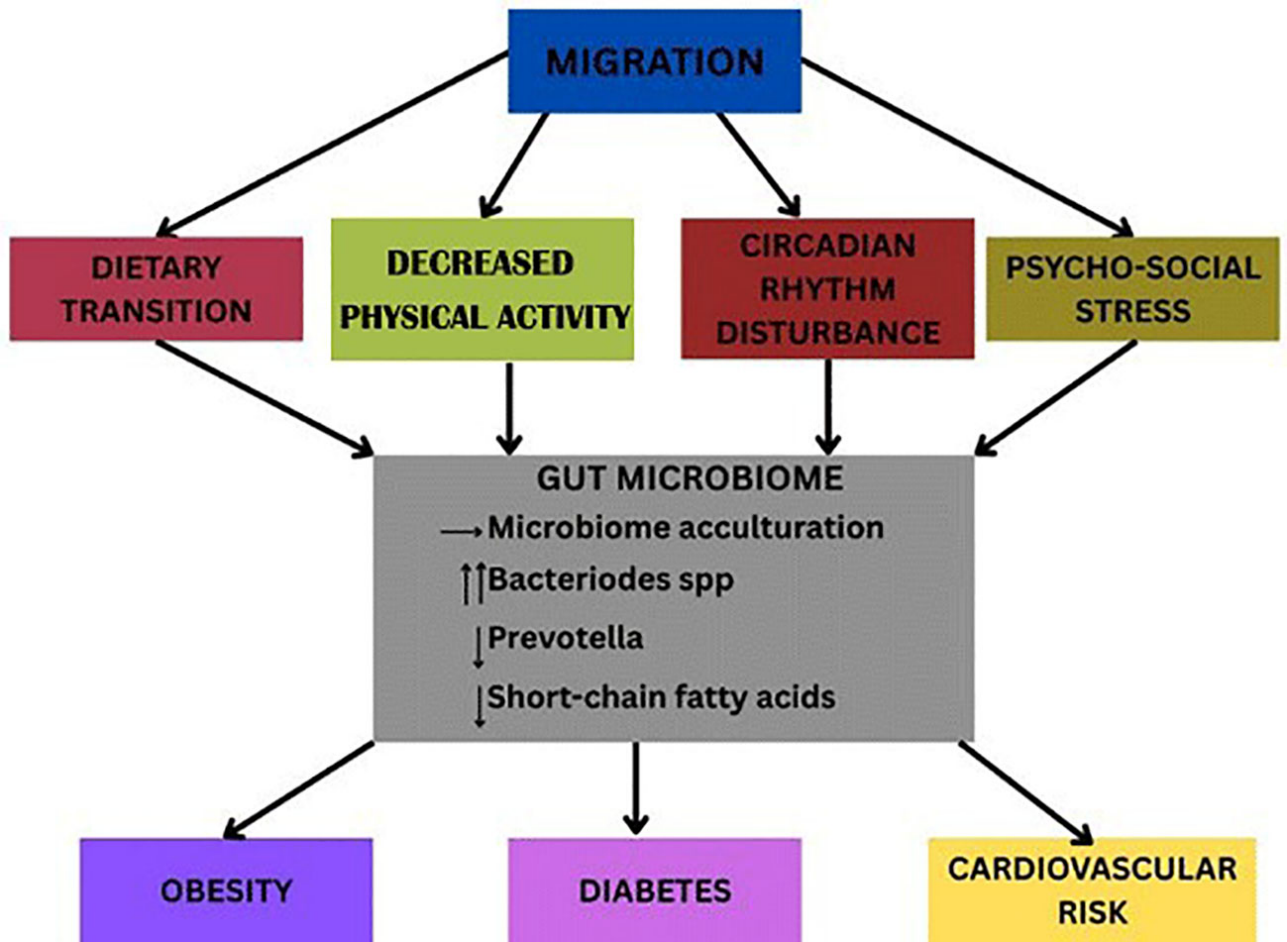
Gut microbiome and weight gain after immigration. Pathways from migration associated with obesity via changes in the microbiome. Exposure to a Western diet and altered routines (circadian disruption; reduced activity) drive dietary shifts and microbial remodeling, reduced diversity, loss of fiber-fermenting Prevotella, and greater Bacteroides abundance. Immigrants adapting to industrialized environments have reductions in short chain fatty acids (SCFAs), increases in low-grade inflammation, and metabolic dysregulation to promote weight gain, insulin resistance, and downstream cardiometabolic effects.

In summary, the circadian rhythm acts as a master regulator that integrates molecular, metabolic, and epigenetic signals to maintain cellular and systemic homeostasis. It ensures that energy utilization, redox balance, and gene expression are synchronized with daily environmental cues. Disruption of this rhythm disturbs these tightly regulated processes, predisposing individuals to weight gain leading to metabolic and cardiovascular diseases, as summarized in **Figure 3**.

### 2.4 Psychosocial stress, acculturation, and gut microbiome dysregulation

Migration introduces a cascade of psychosocial stressors, including language barriers, cultural dissonance, underemployment, and social isolation, that exert both direct and indirect effects on metabolic health (77, 78). Chronic activation of the hypothalamic–pituitary–adrenal (HPA) axis as a result of sustained psychological stress elevates cortisol levels, which enhance hepatic gluconeogenesis, promote visceral fat deposition, and induce insulin resistance (79–81). Dysregulated cortisol rhythms also disrupt appetite-regulating hormones such as ghrelin and leptin, heightening hunger and preference for high-fat, high-sugar foods (82). Over time, these hormonal and behavioral responses create a metabolic environment conducive to obesity and related disorders, particularly when combined with reduced physical activity and poor sleep quality (83).

Emerging evidence suggests that chronic psychological stress also exerts significant influence on gut microbiome composition and function, forming a critical biological bridge between mental distress and metabolic disease. Sustained activation of the HPA-axis increases gut permeability and alters mucosal immunity, allowing stress-induced glucocorticoids and catecholamines to modify microbial balance. This promotes the overgrowth of pathogenic bacteria and reduces populations of beneficial commensals such as *Lactobacillus* and *Bifidobacterium*. Such dysbiosis diminishes SCFA production, weakens intestinal barrier integrity, and heightens systemic inflammation, mechanisms known to contribute to insulin resistance and visceral adiposity. In immigrant populations, where stress often coexists with dietary acculturation and disrupted circadian rhythms, these microbial alterations magnify metabolic vulnerability. Thus, psychosocial stress not only alters eating behavior and hormonal balance but also reshapes the gut ecosystem, reinforcing a vicious cycle of inflammation, oxidative stress, and weight gain.

Addressing these effects requires an integrated strategy combining culturally sensitive mental-health support, social inclusion programs, and microbiome-supportive nutritional interventions (e.g., prebiotic- and probiotic-rich diets) (84). Together, these approaches can mitigate stress-related gut dysbiosis, improve metabolic resilience, and reduce obesity risk among immigrant communities facing chronic psychosocial strain.

### 2.5 The gut microbiome: a central modulator of weight gain following immigration

The U.S. hosts nearly one-fifth of the world’s immigrants, where post-migration obesity risk is elevated, with refugees experiencing sharp increases in BMI (88, 89). Western diet acculturation, food insecurity, and reduced activity contribute, but do not fully explain the excess risk for weight gain (90). Because the microbiome rapidly mirrors new diets and environments (91, 92), it offers a lens on metabolic change. Work following Hmong and Karen migrants *(refugees from South East Asia-{{-}}–Hmong mostly from Vietnam and Karen mostly from Burma)* showed that even brief residence in the U.S. increases the Bacteroides: Prevotella ratio and erodes carbohydrate-active enzyme capacity (15, 93). These patterns reflect “microbiome Westernization” and align with the disappearing microbiota hypothesis (94). Second-generation immigrants often exhibit Western-like microbiomes despite distinct diets, suggesting intergenerational depletion. Collectively, these findings link immigration to durable microbial perturbations that elevate metabolic risk. The gut microbiome acts as a dynamic interface between environmental cues and host metabolism, integrating the effects of diet, physical activity, stress, and circadian rhythm. Together, these factors shape microbial composition, metabolic outputs, and the risk of obesity and metabolic disease.

**Figure 4:**
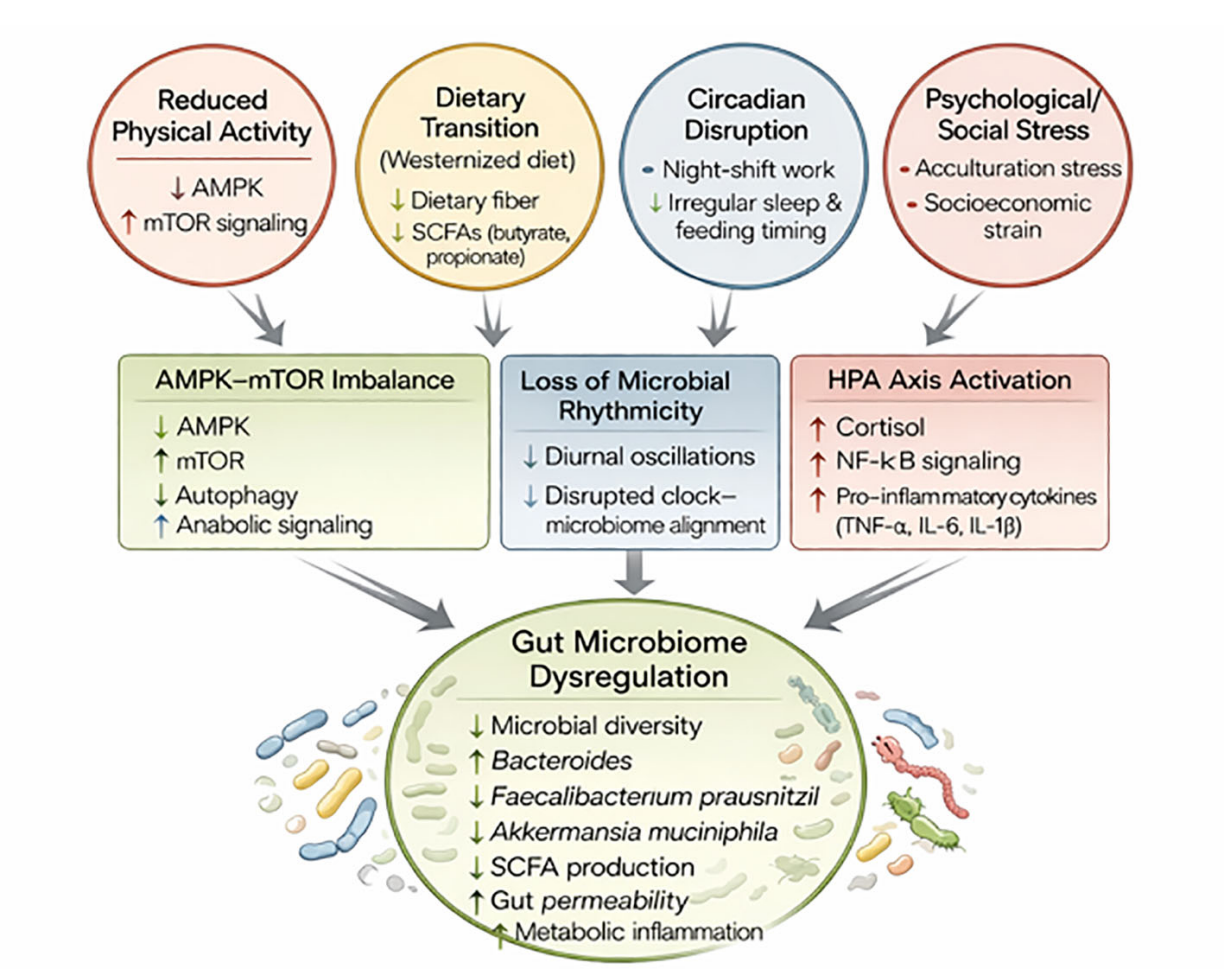
Proposed pathways linking migration-associated lifestyle changes to gut microbiome dysfunction. Migration is frequently accompanied by reduced physical activity, dietary transition to low-fiber Westernized diets, circadian disruption, and heightened psychological stress. Reduced physical activity and dietary fiber intake suppress AMPK signaling and short-chain fatty acid (SCFA) production, promoting pathological activation of mTOR signaling. Circadian misalignment disrupts microbial rhythmicity, while chronic psychosocial stress activates the hypothalamic–pituitary–adrenal (HPA) axis, increasing cortisol and NF-κB–mediated inflammation. These converging pathways drive gut microbiome dysregulation characterized by reduced microbial diversity, depletion of beneficial taxa, impaired gut barrier integrity, and increased metabolic inflammation, thereby contributing to obesity and metabolic disease risk in immigrant populations.

The combined effects of diet, physical activity, psychological stress, and circadian disruption (e.g., night-shift work) on weight gain, particularly among migrant populations, suggests that the gut microbiome may serve as a central mediator linking these factors to metabolic outcomes (95)(**Figure 4**). In immigrants adopting a Western lifestyle, this becomes especially relevant: migration often coincides with dietary acculturation (higher ultra-processed food intake, lower fiber), changes in exercise patterns and activity, elevated psychosocial stress of adaptation, and disrupted sleep/meal timing, all of which converge on the intestinal microbial ecosystem.

When individuals move from non-Western to Westernised environments, their gut microbiota tend to “Westernize” (i.e., lose diversity, shift from Prevotella-rich to Bacteroides-rich profiles) in association with increased adiposity. A seminal study of Southeast Asian immigrants to the U.S. reported that increased duration in the U.S. was associated with reduced gut microbial diversity, concomitant with greater obesity prevalence (15). Mechanistically, these shifts may impair microbial fiber-fermentation capacity, favor energy harvesting, and promote low-grade inflammation—setting the stage for weight gain. Indeed, in a large cohort of U.S. Hispanics/Latinos, longer U.S. residence was associated with reduced gut microbiome diversity and functions of fiber degradation, and these alterations correlated with higher BMI (96).

Furthermore, lifestyle modifiers common to immigrants amplify this microbiome–weight gain axis. Lower physical activity and high adiposity are linked to less beneficial microbial profiles, and exercise favorably modulates gut microbiota composition (97). Chronic psychosocial stress (from migration, acculturation pressure, socioeconomic strain) and circadian disruption (e.g., night-shift work) likewise perturb the gut microbiome. For example, shift work is associated with gut dysbiosis, systemic inflammation, and metabolic dysregulation (95). Thus, the intertwining of altered diet/exercise, psychological stress, and sleep/meal timing disruption in immigrants converges on the microbial ecosystem, helping to explain why immigrant populations often exhibit weight gain and metabolic risk post-migration.

## 3 Limitations

This literature review has several limitations that should be acknowledged: (1) Immigrant populations are highly heterogeneous with respect to ethnicity, cultural practices, socioeconomic status, country of origin, and host environment. These factors can individually or collectively influence dietary patterns, gut microbiome composition, mTOR signaling, and metabolic outcomes (98). As a result, the pathways proposed here may not be uniformly applicable across all migrant groups; (2) Generational effects are not fully captured in the existing literature; first-generation immigrants often exhibit distinct changes in microbiome and metabolic profiles compared with second- and later generation populations, reflecting progressive acculturation, early-life exposures, and epigenetic adaptation over time (99); (3) The substantial interindividual variability in host genetics may modify susceptibility to microbiome remodeling and mTOR activation, can influence insulin sensitivity, adiposity, and inflammatory responses (100); (4) Genetic differences in nutrient sensing, immune regulation, and circadian biology are rarely considered in migration-related metabolic studies; (5) Accurate measurements of key lifestyle exposures remains challenging. Most studies rely on self-reported data on dietary intake, physical activity, and sleep patterns, which are prone to recall bias and misclassification (101, 102). These limitations underscore the need for longitudinal, multi-omics studies integrating host genetics, objective behavioral measures, and microbiome profiling across diverse migrant populations and generations.

## 4 Public health impact

Obesity levels in immigrant populations is increasing and nears native-born levels within one to two decades of arrival (103, 104). As this prevalence grows, so does the burden of diabetes, hypertension, and atherosclerotic disease in immigrant communities (105), likely due to the cumulative effects of biological and structural stressors in obesogenic environments. The economic toll is substantial, encompassing direct medical costs and indirect productivity losses, which disproportionately affect immigrants in labor-intensive and service roles (106).

Reducing this burden requires culturally informed, upstream strategies. Interventions should address night-shift exposure, acculturative stress, food insecurity, and preventive-care access (107–109). City- and community-level programs that make healthy foods and safe recreation accessible—and that respect traditional foodways—are essential (110, 111). Language-appropriate care, mobile health, and expanded coverage can enable earlier screening and management (112). Tackling biological and structural drivers in tandem is critical to bending the curve.

## 5 Conclusion

Migration initiates a complex cascade of biological, environmental, and behavioral transitions that converge on the gut microbiome as an important mediator of metabolic health. The evidence synthesized in this review suggests that changes in diet, physical activity, circadian rhythm, and psychosocial stress collectively remodel the gut microbial ecosystem, transforming a metabolically resilient community into one that promotes inflammation, insulin resistance, and adiposity. The process of “microbiome Westernization” characterized by reduced diversity, depletion of *Prevotella* species, and enrichment of *Bacteroides*, represents a biological signature of acculturation, mirroring the transition from traditional plant-based diets and active lifestyles to sedentary, high-calorie, and low-fiber environments. Night-shift work and irregular meal timing further desynchronize microbial oscillations and metabolic pathways, amplifying the risk of obesity and type 2 diabetes. Thus, the gut microbiome provides an integrative framework that links social and behavioral determinants of migration to molecular mechanisms of metabolic disease. Future efforts should longitudinally track the gut microbiome in recently arrived migrants and follow this with the duration of resettlement, and stratify this data by age, gender and type of employment etc.

Addressing this growing public health challenge requires an interdisciplinary approach that recognizes the microbiome as both a marker and modifiable target of post-migration health. Interventions that restore circadian alignment, promote physical activity, and support access to fiber-rich, culturally familiar diets can reestablish microbial diversity and metabolic balance. Community-based programs must also tackle broader social determinants, such as shift-work exposure, acculturative stress, and food insecurity, that perpetuate metabolic disparities among immigrants. Ultimately, strategies that integrate microbiome-informed nutrition, chronobiology, and social policy hold the greatest promise for preserving metabolic health during the migration process and preventing the erosion of the “healthy immigrant effect” across generations.
